# Priority Setting on the Path to UHC: Time for Stronger Institutions and Stronger Health Systems: Response to Recent Commentaries

**DOI:** 10.15171/ijhpm.2019.39

**Published:** 2019-05-28

**Authors:** Ole F. Norheim

**Affiliations:** Department of Global Public Health and Primary Care, University of Bergen, Bergen, Norway.

In response to my editorial,^1^ I received to my surprise four thoughtful, critical, enthusiastic, and constructive comments and reflections. My original aim was modestly to suggest a Theory of Change (ToC) for translating economic evidence, from the Disease Control Priorities (DCP) projects and similar sources, to better priority setting within countries.

Davis and Walker offer a more comprehensive alternative, putting the client at the center. They suggest developing “a ToC to support progressive institutional development to deliver on universal health coverage [UHC].”^2^ They argue that the desired outcomes must be locally sustainable, multi-functional, learning institutions and better governance of health systems for efficiency and equity.

I could not agree more. Better priority setting is only one of the elements needed for stronger health systems and accountable governance.

Against my view that creating institutions is the responsibility of countries, they see a much larger role for development assistance for health and ask how global institutions can better support national governments in low- and middle-income countries to establish dynamic learning institutions for better governance.

Although global institutions could get their act together and see technical assistance as a much more important part of development assistance for health, I maintain that local capacity strengthening is a necessary first step to create demand for evidence and for national institutions using evidence for priority setting and health systems strengthening.

In a similar vein, Isaranuwatchai and colleagues argue that the best way forward for the global health community is a new platform, a global community of practice, that can coordinate and integrate the many existing development initiatives – such as DCP, iDSI, Global Burden of Disease, Tufts’ Cost-Effective Analysis registry, HIV/TB/malaria modelling consortia, Global Health Costing Consortium, the Joint Learning Network, and so on – “that is driven by countries’ asks.”^3^

I agree that a global community of practice is needed, and I would welcome such a coordinated effort. Yet, again I fail to see that this is a first step in a ToC and that it would put “the client at the center.” Unless each country, and its government institutions, see the need to set priorities through evidence-based approaches, a new platform would mostly benefit researchers and institutions in the United States and Europe.

Against my view that we need to go beyond cost-effectiveness and include distributional concerns and fair process, they argue this new platform should take “Pride in Cost-Effectiveness” and that the world needs more, not less, cost-effectiveness analysis. They also criticize DCP for not using its significant global influence to challenge questionable global norms, especially World Health Organization’s (WHO’s) earlier guidance on willingness-to-pay thresholds that effectively said that only interventions costing more than three times gross domestic product per capita per quality-adjusted life year gained or disability-adjusted life year averted are not cost-effective.

I certainly agree that cost-effectiveness information is key to priority setting, but we need to acknowledge that equity impact, financial risk protection and fair process is as important for public acceptability. Global norms are changing, and I would argue that the strong empirical work of Claxton et al on the opportunity cost of not getting cost-effectiveness thresholds right is gaining traction and will in due time change the views of key actors such as WHO and DCP.^4,5^ The data and the arguments are convincing and the best argument wins in the long run.

Rachel Nugent’s reflections are especially useful for her honest defense of the underlying ToC of DCP3, its achievements, and its shortcomings.^6^ The main target audience for DCP 1-3 was, with its key message that investing in health has high returns, the ministries of finance and similar decision-makers in global health institutions. This indirect effect should not be underestimated and may over time create more demand for priority-setting evidence within global institutions and ministries of health. DCP3 reviewed and summarized high-quality health intervention effectiveness and cost-effectiveness evidence and proposed essential health benefit packages for resource constrained countries as well as for countries aiming to scale up to reach UHC by 2030.^7^

Yet, Nugent admits that DCP3 has not influenced national priority-setting to the extent that participants in DCP3 had hoped. Although Prabhat Jha^8^ cites some excellent early examples of the influence of DCP in India, efforts of this kind can and must aim to translate evidence to better priority setting within countries – not least in the era of Sustainable Development Goals and UHC.

An important observation is that we are already in “a post-DCP3 world.” On-line journals, global resources such as WHO’s Global Health Observatory, World Bank Open Data, and the Global Burden of Disease study make data available almost in real time. At a more technical level, a review of the usefulness of DCP3 results for Malawi’s revision of their essential benefit package revealed that the presentation of main results in book chapters and journal publications without a combined database covering all interventions limited its user-friendliness.^9^ In addition, the authors argue that disaggregated estimates of costs and effects, quantified uncertainty, and a systematic assessment of transferability from one context to another would have made this evidence more useful for decision-makers.

Here is clearly a role for development assistance for health and a global community of practice. Research is a global public good and should be made available for all countries without delay and in a user-friendly way. Neither DCP or WHO-Choice have yet invested enough in making their results available for use and critical scrutiny.

Although Prabhat Jha, in his comment, highlights the achievement of DCP and argues that properly done, “DCP could be as important over the next 25 years as it has been in the past 25 years,”^8^ he also identifies improvements to the DCP approach that are required to meet the changing landscape of global health and to ensure relevance to countries. Most importantly, there is a need for a large costing platform, building upon WHO’s earlier analyses in different settings and regions. These data should be shared openly for unrestricted use on a globally accessible website. I agree, and it seems to me that all the comments converge toward a similar consensus.

Jha also agrees with the need for local capacity building and calls for more reliance on local and direct mortality data rather than on models. Direct mortality data are needed to assess the impact of programs as modelled data cannot separate real changes from changing modelling assumptions. His last point is worth noticing. Local data are needed for local decision-making, monitoring and evaluation.

In summary, I find the comments extremely useful as they converge into a list of improvements of my initial proposed ToC.

First, reformulate the long-term outcomes to not only include institutionalized priority setting, but also health systems strengthening through national, multi-functional, learning institutions with better governance for improved efficiency and equity.

Second, identify capacity strengthening as the key input to create country demand for relevant evidence, systematic priority setting, and accountable governance of health systems that deliver high-quality essential UHC.

Third, identify as another key input a global community of practice that can help generate the needed contextualized evidence, made available in a user-friendly way, with disaggregated information, quantified uncertainty, and a systematic assessment of transferability from one context to another.

Fourth, assign responsibility for establishing this new platform. This should be seen as a key part of development assistance for health and would require coordination and attention from WHO, the World Bank/GFF, GAVI, the Global Fund, the United States Agency for International Development, and others. In my view, natural focal points are WHO and the World Bank as they can identify demand from countries, bring global institutions to the table, coordinate centers of expertise as providers of technical assistance, and facilitate work within countries.

Fifth and finally, forget the idea that technical assistance equals consultants flying in and out of countries on short-term missions. Providing technical assistance means building national capacity for efficient and equitable resource allocation, training people up to PhD level and establishing national academic centers of excellence, learning from comparable countries, and exchange of knowledge, learning, skills, and responsible leadership.

With these elements in place, I believe we together could develop a more ambitious ToC for better priority setting, stronger institutions, and stronger health systems for achieving UHC.

## Ethical issues

Not applicable.

## Competing interests

The Author leads, with Stéphane Verguet, the project Disease Control Priorities – Ethiopia. Funded by Bill and Melinda Gates Foundation.

## Author’s contribution

OFN is the single author of the paper.

## References

[1] Norheim OF (2018). Disease control priorities third edition is published: a theory of change is needed for translating evidence to health policy. Int J Health Policy Manag.

[2] Davis A, Walker DG (2019). On the path to UHC - global evidence must go local to be useful: Comment on “Disease control priorities third edition is published: a theory of change is needed for translating evidence to health policy. ”Int J Health Policy Manag.

[3] Isaranuwatchai W, Li R, Glassman A, Teerawattananon Y, Culye AJ, Chalkidou K (2019). Disease control priorities third edition: time to put a theory of change into practice: Comment on “Disease control priorities third edition is published: a theory of change is needed for translating evidence to health policy. ”Int J Health Policy Manag.

[4] Woods B, Revill P, Sculpher M, Claxton K (2016). Country-level cost-effectiveness thresholds: initial estimates and the need for further research. Value Health.

[5] Claxton K, Martin S, Soares M (2015). Methods for the estimation of the National Institute for Health and Care Excellence cost-effectiveness threshold. Health Technol Assess.

[6] Nugent R (2019). Reflections on Norheim (2018), disease control priorities third edition is published: Comment on “Disease control priorities third edition is published: a theory of change is needed for translating evidence to health policy. ”Int J Health Policy Manag.

[7] Jamison DT, Alwan A, Mock CN (2018). Universal health coverage and intersectoral action for health: key messages from Disease Control Priorities, 3rd edition. Lancet.

[8] Jha P (2019). The future of disease control priorities: Comment on “Disease control priorities third edition is published: a theory of change is needed for translating evidence to health policy. ”Int J Health Policy Manag.

[9] Arnold M, Griffin S, Ochalek J, Revill P, Walker S (2019). A one stop shop for cost-effectiveness evidence? Recommendations for improving Disease Control Priorities. Cost Eff Resour Alloc.

